# Foreign Correspondence

**Published:** 1854-11

**Authors:** Austin Flint

**Affiliations:** Professor of the Theory and Practice of Medicine in the University of Louisville


### ART. II.—FOREIGN CORRESPONDENCE.

BY AUSTIN FLINT, M. D., PROFESSOR OF THE THEORY AND PRACTICE OF MEDICINE IN THE UNIVERSITY OF LOUISVILLE.

DEAR DOCTOR:—

I have already intimated an intention of giving you some account of the St. Louis Hospital. I propose to do so in this letter. This is one of the largest, and oldest of the Parisian hospitals. It was founded by HENRY IV, in 1607, on the site of an alms-house which had existed from a remote period. The prevalence of an epidemic disease called the plague, supposed to be contagious, gave rise to the establishment of this hospital. Owing to the great accumulation of cases in the wards of Hôtel Dieu, patients were placed in the same bed to the number of four, and sometimes even six. The mortality was frightful, and, growing out of the urgent necessity for more accomodations for the relief of the sick, the St. Louis Hospital was commenced, and completed in four years. The name is said to have been given to it in consequence of ST. LOUIS having died, in 1270, with the epidemic disease which prevailed at the time of its construction.

It is situated in the faubourg St. Martin, in the north-eastern part of the city, near the barrier, on the *Rue Bichat*, so called in honor of the founder of the science of general anatomy. Numerous streets in Paris bear the names of those who have been distinguished in medical science. There are the *Rue Dupuytren*, *Rue Larrey*, *Rue Richerand*, etc. Has this slight tribute of respect ever been paid to the memory of an American physician or surgeon?

The edifice originally erected by HENRY IV, still remains, but large additions have been made from time to time; and a new commodious building is just finished. The wards are large and well ventilated, and within the extensive area encircled by a high wall are spacious courts and gardens ornamented with trees and shrubbery.

A separate building is allotted to pay patients, who are charged two francs per day. Patients received gratuitously, affected with diseases which do not altogether compromise their ability to labor, are encouraged to work in the gardens by a premium of one sou for every hour they are thus employed.

The establishment has a large washhouse, and extensive arrangements for baths of which I shall presently give some account.

The sick are nursed by twenty-five sisters of charity of the order of the *Dames de St. Augustin*, who reside in a house contiguous to the walls of the hospital.

Gas for lighting the hospital is manufactured in the vicinity, and here was the first gas apparatus established in Paris.

The St. Louis Hospital is appropriated chiefly to the treatment of diseases of the skin. It is one of the special hospitals. A few patients, however, with other diseases are received, and cases of accidents and surgical affections, occurring in that part of the city in which it is situated, are sent there. The surgical service, for the most part consisting of cases thus derived, is large. During the year 1850 there were admitted 5124 medical, and 2528 surgical patients. Of the former, 136, and of the latter, 189 died. The whole hospital contains 853 beds, and the average number of inmates is from five to six hundred.

During my residence at Paris I have devoted two afternoons weekly to the study of the cases of cutaneous disease at this hospital. The opportunities for clinical observations pertaining to that class of maladies, as would be inferred from the facts already stated, are excellent. Cases illustrative of similar forms of disease are presented in groups, so that the different subdivisions of each class, as well as the different classes of the *cutanei* may be at once compared, without the necessity of waiting for examples to offer themselves at disconnected epochs, as in general hospitals, and still more in private practice. The great advantage of this in acquiring, practically, familiarity with the external characters upon which are based the divisions into order, genera, species and varieties, and verifying the principles of diagnosis, as well as observing the effects of remedies, are sufficiently obvious. The clinical facilities for which Paris is celebrated, consist mainly in its special hospitals; and, among the latter, that of St. Louis claims particularly the attention of those who visit the French metropolis for medical improvement, inasmuch as institutions devoted to cutaneous affections are rare even in large cities. London, which is double the size of Paris, has no extensive skin hospital. The importance of one is, however, beginning to be felt in that city, and a small institution for this class of diseases, as I was informed, has recently been established. It is doubtless owing in a great measure to the want of the facilities afforded by hospitals appropriated to this purpose, that the study of diseases of the skin is so generally neglected by medical practitioners.

The medical staff at St. Louis consists of M. M. CAZENAVE, DEVERGIE, GIBERT, BAZIN, and HARDY. All these names are conspicuously associated in the modern literature of cutaneous affections.

The work by CAZENAVE and SCHEDEL, entitled *Abrégé Pratique des Màladies de la Peau*, has long been a standard treatise, the first edition having been published in 1828. It has passed through several editions, and an English translation, with notes by Dr. BULKLEY, of New York, was issued in this country two or three years ago. An elementary treatise professedly based on the theoretical and clinical teachings of M. CAZENAVE, has appeared quite recently in Paris from the pen of Dr. CHAUSIT. A course of clinical lectures by M. CAZENAVE was delivered during the past summer, which was well attended. His service is more numerously followed than that of either of his colleagues, and he appears to be esteemed as the highest authority in Paris on all that pertains to his department. In some respects, however, he shows a disposition to adhere to the past with an obstinacy which prevents him from appreciating the evidence in favor of newly developed facts. He contends strenuously against the reality of the discovery of *cryptogamous* productions in certain of the eruptions peculiar to the face and scalp. He repudiates in toto, the results of the microscopical observations of BAZIN, ROBIN, and others, insisting that they are mistaken—that their conclusions are due to the fallacies incident to the use of the microscope. In this course he is sustained by few of those who, in general, place confidence in his opinions.*

* M. CAZENAVE has in process of publication a magnificent series of plates illustrative of skin diseases. About half the series are already issued. They are accompanied by text descriptive of the affections figured.

M. DEVERGIE, has long been one of the attending physicians at St. Louis, having been the successor of BIETT. He gives clinical lectures, and his service is respectably attended. He has, during the present year, published a treatise entitled *Traité Pratique des Máladies de la Peau*, in which he dissents, in several particulars, from views inculcated in the work by CAZENAVE and SCHEDEL. M. DEVERGIE observes for himself, and is an independent thinker. His appearance and deportment in his wards are well calculated to inspire confidence in his honesty and judgment. My visits at the hospital were oftener in his wards than in those of CAZENAVE, and after a careful perusal of his work, I am disposed to think that it will prove a valuable addition to the literature of this department of practical medicine.

M. BAZIN has rendered valuable service in pathological investigations pertaining to skin diseases by his researches on the nature and treatment of the forms of eruption characterized by the presence of cryptogamous plants. He classes all the affections thus characterized under the head of *teignes*. He published a small treatise on the subject in 1853. He is also distinguished for having introduced into the practice at St. Louis Hospital a more expeditious mode of curing *scabies* than had previously been employed. This method I shall describe presently.

M. HARDY, at the present time, has charge of the itch department of the hospital, and has improved the method of effecting rapid cures introduced by M. BAZIN to which I have just alluded.

The method of treating scabies, the introduction of which at St. Louis is due to M. BAZIN, consisted in first employing baths, and forcible friction with soap, in order to cleanse the skin, and rupture the vesicles; afterward, friction three times daily, for two successive days, were made with the *Pommade d'Helmerick*, which is composed of sulphur, the carbonate of potash and lard.* The latter frictions ended, another bath, with soap, was employed, and the patients were discharged cured in three days.

The plan at present pursued, as perfected by M. HARDY, is as follows:—

The patients first apply, with friction, *savon noir* (soft soap) and

* Carbonate of potash, one part; sulphur, two parts; lard, eight parts, (by weight).

take a warm bath, which occupy an hour. Next they continue friction with the *pommade* of sulphur and carbonate of potash for half an hour. An alkaline bath comes next in order, lasting half an hour. The whole process consumes two hours, and they are at once discharged cured. They are, however, requested to return at the next day appointed for external consultation for cases of this description, in order to be examined, and if the cure be not complete, the process is repeated. I was assured that five of every six cases are effectually cured by one process. Other eruptions, however, eczema, impetigo, and lichen, succeed in a considerable proportion of cases.

The exhibition of the application of this method of cure to a large number of cases collectively, is one of the medical curiosities of Paris, which should not be overlooked more than the operations with the hot irons by JOBERT. Two days in the week are designated for persons to apply for treatment. At the time I was present, there were from thirty to forty candidates. Sufficient accomodations were provided for the whole number to bathe simultaneously. After the bathing, they were marched into a room by an official in uniform, who described to them the details of the stage of the process which was next to take place. On the floor was a large *tumulus* of the *pommade.* They were directed to take, each a certain quantity, and to rub all the accessible parts of the body, using forcible friction; they were to apply the *pommade* to the back for each other; the frictions were to be continued for half an hour. The comical exhibition of from thirty to forty naked persons carrying into active execution the foregoing instructions, can be better imagined than described. A few of the patients were children too young to make the applications for themselves. In these cases the duty was performed by assistants. The children gave evidence of the severity of the frictions by loud lamentations, and some of the men were unable to sustain the smarting with perfect stoicism. The half hour expired, an alkaline bath followed, and the process was finished.

This treatment is based on the doctrine that the disease is dependent on the presence of the living *acarus.* The object is to uncover the insect, force it from the burrow, and destroy it by means of the sulphur, alkali and lard, and, also, the germs of a future offspring. This doctrine, however, is not accredited by all. M. DEVERGIE, for example, regards the production of the *acarus* as merely incidental to the progress of the disease, not the efficient cause of the disease. He would account for the cure by the measures just described, not solely because the acarus is destroyed, but from the medicinal efficacy of the remedies employed, irrespective of this effect. In his opinion the measures are needlessly active and rough; and injudicious in consequence of the subsequent eruptions to which they frequently give rise.

The history of the discovery of the existence of the *acarus scabii* is connected with a curious imposition practiced at St. Louis. An Arabian physician first noticed the presence of an insect in connection with the itch-vesicles, but the fact excited little or no attention, and was almost forgotten until the sixteenth century, when it was described by HAUPTMANN. It was described more fully by CESTONI in 1687. ALIBERT and BIETT, however, the distinguished physicians at St. Louis, failing to verify its presence, were led to deny the fact of its existence. The opinion of these writers was regarded as such high authority, that, in France, the co-existence of an insect with scabies was generally either distrusted, or disbelieved; when a person named Galès (a name singularly resembling that of the disease in French, viz: *gale*) professed to demonstrate the fact of its presence, by adroitly producing the *mites* from cheese which he pretended were extracted from the itch-vesicles. He practiced the fraud with such success, that the Academy of Medicine, having appointed a commission to examine the subject, and witness the process of extraction, were completely deceived, and distinguished honors were conferred on M. Galès. The subsequent discovery of the imposition tended to render the existence of the true *acarus* more doubted than before, but at length, the fact was established beyond farther question by M. RENUCCI, M. ALBIN GRAS, and M. RASPAIL in 1834–5, and M. BOURGUIGNON in 1843.*

I witnessed the extraction of several *acari* by M. FREMINEAU, a young physician of great promise, one of the *internes* at St. Louis. Vesicles were selected which were recent, and presented a well marked furrow. Introducing the needle at the extremity of the

* These statements are quoted from the work by M. DEVERGIE.

furrow and withdrawing it, a small opaque body was apparent on the end of the needle, which, placed on paper, gave evidence of animation to the naked eye, but more clearly with a common pocket magnifying glass. Dr. WILSON states that the animal seizes hold of the needle, and is thus brought out. This may be doubted. The cuticle at the end of the furrow being gently raised, if the *acarus* be there, it forms a free body in so small a space, that it is easily, as it were, *scooped up* with the instrument. M. FREMINEAU easily caught one at the first trial, and very little time and trouble were required to collect a number for subsequent microscopical examination.

The present mode of treating scabies has been of great utility to the hospital. Formerly patients with this affection were admitted into the wards, and constituted a large proportion of the cases in the hospital. Now they do not enter the hospital, and hence, the accommodations for other cases are proportionably greater than they were.

A vast number of out-patients are examined and prescribed for at the St. Louis Hospital. For this purpose the medical officers attend at the consultation room daily, in rotation. For these cases as well as for the patients in the hospital, extensive bathing accommodations are provided, and a large proportion of the patients who apply, receive only permits for simple, or medicated baths. The number of baths administered is immense, viz: in one year 50,000, exclusive of 40,000 fumigations, and 2,000 *douches.*

The following description of the provisions for bathing, etc., at St. Louis, is copied from notes written after a visit made expressly to examine this department of the institution: In a long ward on the ground-floor, are thirty bathing tubs, arranged in two rows, with provision for the supply of cold and warm water in any quantity. These are appropriated every Wednesday and Saturday, in the forenoon, by patients who undergo the process for the cure of scabies.

Adjoining this ward is a small apartment containing accommodations for two baths, for isolated cases.

An apartment is devoted to *douches.* A metal tube is connected, by means of a long flexible hose, to a spout regulated by a stop-cock, allowing a stream of water to be directed, at different distances, on any part of the body. There are arrangements for *douches* with warm or cold water in this apartment. In another room is a chair constructed for the administration of *lavements*.

Passing into another apartment, a metal cylinder about two feet in diameter, extends from the stone floor, vertically, about two feet and a half. This room is small, the walls, as well as the floor, is of stone, and a series of stone steps, constructed in the form of a semicircle, extending upward about ten feet. This apartment is for vapor baths. The patient taking different altitudes by means of the stone steps, can vary, to some extent, the degree of exposure to the vapor.

In another room shower baths are administered, the stream of water being subdivided into a great number of jets.

Lastly there is an apartment for fumigations. These are administered by placing the patient within a box containing a series of chairs. The box is tightly closed, with the exception of holes in the top through which the head is passed, leaving the body within the box. The fumes of the medicinal substance employed are produced by heat, conducted within the box, and in this way, brought into contact with the body.

The different kinds of baths, douches, and fumigations prescribed at the hospital, are as follows:—

1. Simple bath.
2. Vapor bath.
3. Sulphurous } 1. Strong.
4. Alkaline } 2. Ordinary.
5. Starch bath, (*de fecule de pommes de terre*).

The last mentioned is of two forms, viz: 1. strong, 2. ordinary.

DOUCHES.

1. Sulphurous.
2. Vapor.
3. Alkaline.

FUMIGATIONS.

1. Sulphurous.
2. Mercurial.
3. Aromatic.

A valuable feature of the practical work by DEVERGIE is that considerable space is devoted to the consideration of baths in connection with the management of cutaneous diseases. Much importance is attached to the employment of the different kinds of baths, by the French practitioners, in the treatment of diseases generally, as well as those seated on the cutaneous surface. They employ them to a much greater extent, both in private, and public practice, than is the custom with us. In this respect, it is probable that American practice offers room for improvement; and, if my leisure permitted, I would append to this letter a translation of that portion of the work just referred to, which treats of this subject. I may find time to do this hereafter; meanwhile, I remain,

Very truly yours,
AUSTIN FLINT.

